# Social anxiety and smartphone addiction among college students: the mediating role of loneliness

**DOI:** 10.3389/fpsyt.2025.1621900

**Published:** 2025-07-23

**Authors:** Xingping Zhou, Baoan Feng

**Affiliations:** College of Teacher Education, Quzhou University, Quzhou, China

**Keywords:** smartphone addiction, social anxiety, loneliness, cross-sectional design, college students

## Abstract

**Background and aims:**

As one of the by-products of smartphone proliferation, smartphone addiction, has negatively affected college students’ academics and well-being, making it a critical issue for educators to address. This study explored how social anxiety and loneliness predict smartphone addiction, adding to prior research in this area.

**Design, setting and participants:**

A cross-sectional research design and a random sampling method were employed to collect data from 2,113 Chinese college students in February 2025. The average age of participants was 19.9 ± 1.23 years (age range: 18–25 years).

**Measurements:**

All participants provided their data on demographic characteristics, social anxiety (assessed using the Revised Social Anxiety Subscale of the Self-Consciousness Scale), smartphone addiction (measured via the Cell Phone Addiction Scale), and loneliness (evaluated with the 6-item De Jong Gierveld Loneliness Scale). Mediation analyses were conducted using Hayes’ PROCESS macro (v4.1) in SPSS (v24). Specifically, Model 4 implemented 5,000 bootstrap resampling repetitions to calculate indirect effects, deriving 95% bias-corrected confidence intervals through percentile-based resampling.

**Findings:**

Statistical analyses showed that social anxiety was positively correlated with loneliness (*r* = 0.269, *p* < 0.001), smartphone addiction (*r* = 0.158, *p* < 0.001), and gender (*r* = 0.058, *p* < 0.01), and loneliness was positively correlated with smartphone addiction (*r* = 0.246, *p* < 0.001) and age (*r* = 0.046, *p* < 0.05). Social anxiety predicted smartphone addiction (*β* = 0.309, *p* < 0.001, *95%CI* = [0.222, 0.396]), and loneliness predicted smartphone addiction (*β* = 0.406, *p* < 0.001, *95%CI* = [0.222, 0.396]), with loneliness partially mediating their relationship (effect = 0.123, *95%CI* = [0.092, 0.157]).

**Conclusions:**

Social anxiety is significantly correlated with smartphone addiction, and loneliness partially mediating their relationship. Reducing loneliness can prevent smartphone addiction among college students with social anxiety.

## Introduction

With the rapid expansion of smartphone functions, improved performance, and declining costs, smartphones have quickly become widespread in China. According to the latest CNNIC (1) survey, smartphones account for 99.7% of internet access among China’s 1.108 billion mobile users. In China, smartphones have become deeply integrated into daily life, with people frequently using them during routine activities — from commuting and household chores to moments before sleep (2). While they offer significant convenience across domains like learning, work, and daily functioning, growing dependence on smartphones has raised concerns. Smartphone addiction — also referred to as problematic smartphone use (3), mobile phone dependence (4), or nomophobia (5) — is characterized by excessive and uncontrolled smartphone use that disrupts an individual’s ability to self-regulate (6). Research indicated that smartphone addiction leads to sleep disorders, postural problems, shoulder pain, increased loneliness, decreased life satisfaction, and poor academic performance (7–10). In view of the far-reaching consequences of smartphone addiction, scholars have recently investigated the association among social anxiety, loneliness, and smartphone addiction during their exploration of risk factors. Current research has confirmed the impact of social anxiety and loneliness on smartphone addiction, yet several research gaps remain. First, while Darcin et al. (11) identified social phobia and loneliness as significant predictors of smartphone addiction among Turkish university students, their study did not explore the mediating mechanisms between variables, and cultural contextual differences may limit the generalizability of their findings. Sun et al. (12) focused on Chinese adolescents and revealed the serial mediating effects of social anxiety and loneliness in the relationship between psychological need satisfaction and smartphone addiction, but failed to address the specific developmental characteristics of university students, as did Lee et al.'s (13) study. Jiang et al. (14) conducted a cross-sectional survey among Chinese nursing students and validated the mediating role of loneliness in the relationship between social anxiety and smartphone addiction, but the specialized sample restricted its general applicability to ordinary university students. Additionally, although Zhao et al. (15) identified both independent and serial mediating effects of loneliness and social anxiety in the relationship between smartphone addiction and interpersonal problems among university students, their research did not systematically analyze how social anxiety and loneliness influence smartphone addiction. Therefore, it is necessary to obtain a sample of Chinese university students covering liberal arts and science students as well as different educational levels through random sampling, systematically investigate the influence of social anxiety and loneliness on smartphone addiction, fill the empirical gap in the relationship between variables among ordinary Chinese university students, and provide a more detailed and targeted theoretical perspective for understanding the mechanism of smartphone addiction among university students.

### Theoretical framework

The most prominent theoretical framework for explaining smartphone addiction was the Interaction of Person-Affect-Cognition-Execution (I-PACE) model (16, 17), which has been widely applied in investigating the causal pathways of smartphone addiction (18, 19). The I-PACE model provides a structured conceptual framework that categorizes the key drivers of maladaptive online behavior into three core dimensions: i) predisposing individual characteristics (e.g., personality traits, fundamental belief systems), ii) cognitive-emotional mechanisms (e.g., maladaptive coping strategies, attentional biases, emotional dysregulation, and stress reactivity), and iii) executive function deficit (e.g., impaired impulse regulation, compromised judgment processes). Within this framework, predisposing variables constitute vulnerability factors that may predispose individuals to develop preferential engagement with specific digital platforms. The cognitive-emotional domain is theorized to mediate or moderate the pathway between these predisposing factors and the emergence of excessive usage patterns (e.g., maladaptive online behavior, addictive behavior). The executive function deficit is a negative consequence of predisposing individual characteristics mediated by cognitive-emotional mechanisms, manifested as specific problem behaviors (e.g., maladaptive online behavior, addictive behavior). Furthermore, the I-PACE model emphasizes the dynamic interplay between these dimensions. Predisposing factors may influence cognitive-emotional mechanisms, which in turn can affect executive functioning. Notably, the model positions social anxiety as a foundational predisposing characteristic within the individual vulnerability cluster. Concurrently, loneliness is conceptualized as an element of the cognitive-emotional response system, potentially operating through coping-related mechanisms. In the I-PACE model, smartphone addiction is considered as one of the manifestations of executive dysfunction. This hierarchical organization emphasizes the dynamic interplay between enduring psychological dispositions and transient psychological processes in the development of digital media overuse.

### Social anxiety and smartphone addiction

Social anxiety, also known as social phobia, is a common psychological disorder (20). Individuals with social anxiety typically exhibit a range of physical anxiety symptoms (such as blushing, trembling, increased sweating, and faster heart rate) and psychological symptoms (such as excessive fear, anxiety, and withdrawal) before or during social interactions (21, 22). Social anxiety is very common among college students (23). A study on 5896 Saudi medical students found that 51% of medical students suffer from social anxiety disorder (20). The negative consequences and impact mechanisms of social anxiety among college students have become one of the important topics of concern for researchers (23–25). Within the vigilance-avoidance paradigm (26), those prone to social anxiety exhibit initial hypervigilance to rejection threats in social-evaluative situations, primarily due to perceived social incompetence and catastrophic appraisals of others’ judgments. This pattern is particularly pronounced during encounters with strangers in novel environments (26). This condition reflects a maladaptive cognitive-behavioral pattern where perceived social inefficacy intersects with hypersensitivity to potential social evaluation across present or imagined interactive scenarios, which may lead to a series of negative consequences. Multiple studies have shown that social anxiety increases the risk of addictive behavior (27–29). Meta-analytic findings reveal that the severity of social anxiety symptoms predicts compulsive smartphone engagement (30–32), and is a driving force behind smartphone addiction (33). Individuals suffering from social anxiety disorder exhibit heightened attentional bias toward negative self-representations during social encounters, and seek out safe social approaches and behaviors (22). Digital communication serves as a functional mechanism for socially anxious individuals to manage interpersonal stressors, offering a regulated platform for social engagement that reduces reliance on direct physical interactions (34, 35). Severe social anxiety disorder patients exhibit a systematic preference for digital communication modalities when avoiding in-person social interactions (36), and this preference is also evident among those with smartphone addiction (37). When compelled to engage in interpersonal interactions, to avoid the nervousness and anxiety caused by such encounters, people may become more reliant on, or even overuse, their smartphones. According to Billieuex et al. (38), anxiety disorders constitute a predisposing vulnerability factor in the developmental trajectory of smartphone-related addictive behaviors. Hence, hypothesis 1 was proposed as follows:

**Hypothesis 1:** *Social anxiety positively predicts college students’ smartphone addiction*.

### Loneliness as a mediator

This study is particularly interested in whether and how loneliness acts as a critical mediator in the causal processes of smartphone addiction. Loneliness is a negative emotional experience, which is defined as individuals’ dissatisfaction arising from a perceived discrepancy between the actual quality or quantity of their social relationships and their desired or expected level of social connection (39, 40). Loneliness, as one of the issues in the field of public health, has received widespread attention from the public in recent decades due to its prevalence among different age groups (41–44). Loneliness may exist throughout an individual’s life, but it is more common in the adolescent population (45, 46). Preliminary findings indicated that college students felt much lonelier than most other age groups (47, 48). College students who left their families and close friends to live and study at university for extended periods reported significant levels of loneliness, over half the cohort (56.7%) met thresholds for clinically significant moderate loneliness, compounded by nearly one-quarter (23.6%) reaching severe diagnostic criteria (49). A recent study indicated that the level of loneliness among young people aged 18-29 showed a linear increase between 1976 and 2019 (50). Previous studies have found that there are many reasons for loneliness among college students, including social isolation during the COVID-19 epidemic (49), growth mindset (51), self-stigma (52). Empirical evidence has established social anxiety as a central factor in predicting loneliness (44, 53, 54). Severe social anxiety symptoms are strongly associated with elevated loneliness levels (55). Loneliness is an unpleasant and even painful subjective experience, which is a risk factor for several negative behaviors and consequences (e.g., suicidal behavior, alcohol-related outcomes, behavioral addiction) (56–58). Individuals may indulge in excessive use of smartphones to cope with loneliness (59). Within the compensatory internet use framework, perceived social isolation is proposed to prompt excessive online behavior as an adaptive-avoidant mechanism for emotional regulation (60). This theory additionally points out that although virtual stimulation has positive effects, the negative consequences (e.g., smartphone addiction) should be paid additional attention. Based on the above findings, we put forward the second hypothesis:

**Hypothesis 2:** *Loneliness would act as a mediator in the relationship between social anxiety and smartphone addiction*.

**Figure 1:**
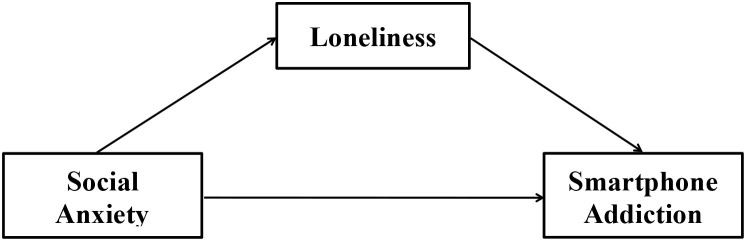
Conceptual model based on the two hypothesis.

Understanding smartphone addiction patterns among China’s college population is critical for developing evidence-based interventions amid rapid digitalization. Against this backdrop, this study, grounded in the I-PACE model, had twofold objectives: First, this research seeks to clarify loneliness as a mediator in the social anxiety-smartphone addiction pathway, filling gaps in current scholarship. Second, the results are anticipated to deepen mechanistic insights into these constructs and guide the implementation of targeted interventions for student mental health. The present study proposes a conceptual model based on the two hypothesis (**Figure 1**).

## Method

### Participants and procedure

Using a cross-sectional design and a random sampling method, this study collected data by distributing digital self-report questionnaires to the target population. The digital questionnaire comprised demographic items and three standardized scales, distributed online via WJX Platform (https://www.wjx.cn/) to Chinese college students between June and July 2024. Inclusion criteria: (1) current students enrolled in three-year vocational colleges or four-year bachelor’s degree programs at Chinese universities; (2) ownership of a personal smartphone with daily usage; (3) provision of written informed consent via digital platforms. Exclusion criteria: (1) non-degree candidates (e.g., exchange scholars, continuing education registrants); (2) questionnaire completion time < 33% of the mean duration; (3) documented diagnosis of psychotic disorders (ICD-10 codes F20-F31) within the past two years.

According to Hair et al. (61), who recommended a sample size of 5 to 20 times the number of questionnaire items, this study’s questionnaire contains 16 items, yielding a minimum sample size requirement of 80 to 320. To account for potential losses from non-response and data cleaning (e.g., removal of invalid responses), and anticipating an effective response rate of approximately 30%, we distributed 2,250 questionnaires to ensure the final valid sample size would be ≥ 650. This approach ensures the robustness of factor analysis and reliability-validity testing.

From an initial cohort of 2,250 participants, 2,113 met the inclusion criteria and were enrolled in the study, resulting in an eligibility rate of 93.9%. Among the participants, the age range was 18 to 25 years, with a mean age of 19.9 years (standard deviation = 1.23), 1,502 (71.1%) participants were female, 637 (30.1%) participants were freshmen, 903 (42.7%) participants were sophomores, 418 (19.8%) participants were juniors, and 155 (7.3%) participants were senior students. 1098 (52.0%) participants were liberal art students and 1015 (48.0%) participants were science and engineering students. Smartphone usage duration analysis revealed: >6 hours/day: 49.2% (n=1,040); 3–6 hours/day: 42.5% (n=897); 1–3 hours/day: 7.8% (n=164); <1 hour/day: 0.6% (n=12). Regarding smartphone usage purposes: online shopping: 93.9% (n=1,985); mobile gaming: 70.7% (n=1,496); entertainment (photography, music/video streaming, microblogging): 97.0% (n=2,050); academic/professional activities (e-learning, information retrieval, work tasks): 91.3% (n=1,931); social communication (voice calls, WeChat interactions): 97.4% (n=2,060).

### Ethics statement

This research was approved by the Institutional Review Board on 17-Jan-2024 (Ethics Reference: 2024011710). Each participant provided an electronic informed consent before enrollment in the study, in compliance with the Declaration of Helsinki. Participants retained full rights to discontinue their involvement at any time without penalty, as outlined in the approved research protocol. Anonymity and confidentiality were strictly maintained. All original data were encrypted and securely stored to prevent unauthorized access, in full compliance with applicable ethical and data protection guidelines. Participants were encouraged to respond as honestly and accurately as possible.

### Measures

#### Cell phone addiction scale

This scale was developed by Roberts et al. (62) to measure smartphone addiction. The 4-item instrument (e.g., “I get agitated when my cell phone is not in sight”) operationalizes smartphone addiction through a 7-point behavioral gradient (1 = *complete non-endorsement* to 7 = *absolute concordance*), ascending mean scores index an increase in the degree of smartphone addiction. The Chinese version demonstrated adequate psychometric validity (63). In this study, the Chinese version scale of the CPAS achieved satisfactory internal consistency (α = 0.78).

#### The revised social anxiety sub-scale of the self-consciousness scale

Social anxiety was measured using the R-SASS-CS, which was originally developed by Fenigstein et al. (64) and subsequently revised by Scheier and Carver (65). This six-item instrument, exemplified by statements such as “I feel anxious when I speak in front of a group,” is scored on a four-point behavioral gradient, where 1 represents “*not at all like me*” and 4 represents “*very much like me*”, ascending mean scores indicate increasing levels of social anxiety. A number of researchers have demonstrated the good validity of the R-SASS-CS when it is applied to the Chinese population (66, 67). Internal consistency for the Chinese version scale of the R-SASS-CS was acceptable in the current sample (α = 0.75).

#### 6-item De Jong Gierveld loneliness scale

Loneliness among Chinese college students was assessed using the DJGLS-6 (68). This bifactorial instrument measures: Emotional loneliness (items 1-3; e.g., “I experience a general sense of emptiness”); Social loneliness (items 4-6, reverse-scored; e.g., “There are enough people I feel close to”). Employing a five-point behavioral gradient, where 1 represents “*never*” and 5 represents “*consistently*”, elevated composite scores reflect greater perceived social-emotional isolation. This scale’s Chinese adaptation has been empirically validated for use in Chinese populations (69). In the present study, the internal consistency coefficients of the two-factor structure in this Chinese version of the DJGLS-6 were 0.67 and 0.88, meeting conventional reliability thresholds.

### Statistical analysis

Statistical analyses were conducted using IBM SPSS (version 24.0) and AMOS (version 29.0). Methodological rigor was ensured through: (1) Harman’s single-factor test for common method biases assessment (70); (2) variance inflation factor (VIF) diagnostics (threshold < 5) via multiple linear regression for multicollinearity evaluation (71); (3) confirmatory factor analysis (CFA) with robust maximum likelihood estimation, evaluating model fit using CFI ≥ 0.95, TLI ≥ 0.95, RMSEA ≤ 0.060 (72). Then, descriptive statistics and correlation analysis were carried out. Finally, this study used Hayes’ (2013) PROCESS macro (v 4.1; Model 4) to test theoretical models of loneliness as a mediator of the social anxiety-smartphone addiction association. The mediation effect was tested using 5,000 bias-corrected bootstrap resamples (73), with statistical significance determined by 95% confidence intervals excluding zero. Smartphone addiction correlates significantly with gender and age, according to previous studies (18, 74, 75). Thus, analyses controlled for gender and age based on established demographic correlations.

## Results

### Preliminary analyses

Prior to formal statistical analysis, Following Podsakoff et al.’s (70) recommendations, we implemented Harman’s single-factor test as a procedural remedy for potential method bias. Using the Kaiser-Guttman criterion (eigenvalues >1), we extracted four principal components. The first unrotated component accounted for 27.52% of total variance, significantly below the 40% threshold suggestive of common method bias (70). To evaluate potential multicollinearity among the three variables, multiple regression analysis was conducted to assess VIF values. Regression diagnostics revealed acceptable VIF levels (max = 1.078), substantially lower than the conservative benchmark of 5 (76), confirming the absence of significant multicollinearity.

Using AMOS 29.0, confirmatory factor analysis (CFA) was performed to assess three competing structural equation models: a one-factor model, a two-factor model, and a three-factor model. The corresponding model fit indices are presented in **Table 1**. Evaluation against established fit criteria (e.g., CFI > 0.90, RMSEA < 0.08; 77) revealed that the three-factor model exhibited significantly superior fit across all reported indices (χ^2^/df = 2.603, *p* < 0.001, RMSEA = 0.028, GFI = 0.989, CFI = 0.992, IFI = 0.992, TLI = 0.987, NFI = 0.987, and RFI = 0.979). These findings support the three-factor structure as providing a more accurate representation of the underlying relationships among the variables, thereby enhancing the model’s explanatory power and robustness (78).

**Table 1:** Fit indices of one-factor, two-factor, and three-factor structural equation models.

Model	*χ^2^*	*χ^2^/df*	*GFI*	*CFI*	*IFI*	*TFI*	*NFI*	*RFI*	*RMSEA*
One-factor model Combining social anxiety, loneliness, and smartphone addiction	8149.753	78.363	0.645	0.446	0.447	0.361	0.444	0.358	0.191
Two-factor model Combining social anxiety and loneliness	5789.675	56.210	0.734	0.609	0.609	0.544	0.605	0.540	0.162
Three-factor model Social anxiety, loneliness, and smartphone addiction	184.778	2.603	0.989	0.992	0.992	0.987	0.987	0.979	0.028

**Table 2** outlines the statistical findings and the relationships among the variables. Social anxiety was positively correlated with loneliness, smartphone addiction, and gender, and loneliness was positively correlated with smartphone addiction and age.

**Table 2:** Distributional characteristics and bivariate associations among core study variables.

Variables	Reliability	Validity	M ± SD	Social anxiety	Loneliness	Smartphone addiction	Gender	Age
Social anxiety	0.75	0.84	2.47 ± 0.59	1				
Loneliness	0.67 and 0.88	0.72	2.70 ± 0.67	0.269***	1			
Smartphoneaddiction	0.78	0.71	4.56 ± 1.25	0.158***	0.246***	1		
Gender^a^			1.71 ± 0.45	0.058**	0.015	0.197***	1	
Age			19.90 ± 1.23	0.003	0.046*	0.033	- 0.077***	1

*N* = 2,113. M, mean; SD, standard deviation. *p < 0.05, **p < 0.01, ***p < 0.001. Gender: male = 1, female = 2.

### Testing for the mediation model

To examine the mediating role of loneliness in the relationship between social anxiety and smartphone addiction, using Model 4 from the PROCESS macro (v 4.1) software plugin for SPSS, developed by Hayes (79), controlling for age and gender. Preliminary analysis revealed a significant direct pathway from social anxiety to smartphone addiction (*β* = 0.309, *p* < 0.001) without the mediator. However, when loneliness was introduced as a mediator, social anxiety exerted a significant positive effect on loneliness (*β* = 0.302, *p* < 0.001), which in turn significantly predicted smartphone addiction (*β* = 0.406, *p* < 0.001). Social anxiety retained a statistically significant, albeit diminished, direct influence on smartphone addiction (*β* = 0.186, *p* < 0.001), suggesting partial mediation by loneliness (see **Table 3** for detailed data).

**Table 3:** Bootstrap mediation analysis: Indirect effect of loneliness on the social anxiety – smartphone addiction association.

Predictors	Loneliness			Smartphone addiction		
	β	SE	95%CI	β	SE	95%CI
Gender	0.004	0.031	[- 0.056, 0.065]	0.525***	0.057	[0.413, 0.636]
Age	0.025*	0.011	[0.002, 0.047]	0.038	0.021	[– 0.003, 0.079]
Social anxiety	0.302***	0.024	[0.256, 0.349]	0.186***	0.045	[0.098, 0.274]
Loneliness				0.406***	0.040	[0.328, 0.485]
R²	0.075			0.106		
F	56.691***			62.586***		

*N* = 2,113. 5000 bootstrap samples. SE, standard error; CI, confidence interval. *p < 0.05, ***p < 0.001.

**Figure 2:**
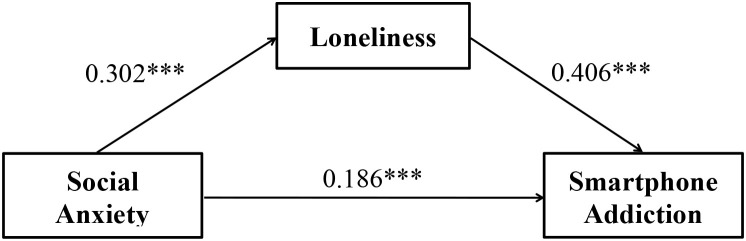
Path coefficients for the mediation model.

Furthermore, as presented in **Table 4**, bootstrap mediation analysis with 5,000 resamples demonstrated a statistically significant indirect effect of loneliness (effect = 0.123, *95%CI* = [0.092, 0.157]) and a persistent direct effect of social anxiety (*β* = 0.186, *95%CI* = [0.098, 0.274]). Loneliness mediated 39.8% of the total effect, indicating partial mediation in the social anxiety – smartphone addiction relationship (see **Figure 2**).

**Table 4:** Bootstrapped effects and 95% confidence intervals.

Effect type	Estimated effect	SE	95%CI	Ratio to total effect
Total effect	0.309	0.044	[0.222, 0.396]	
Direct effect	0.186	0.045	[0.098, 0.274]	60.194%
Indirect effect	0.123	0.017	[0.092, 0.157]	39.806%

*N* = 2,113. Based on 5,000 bootstrap samples. SE: standard error; CI: confidence interval.

## Discussion

This study utilized a sample of Chinese college students to investigate whether loneliness mediates the relationship between social anxiety and smartphone addiction through a structural equation modeling approach. The findings contribute to the literature by elucidating the psychological mechanisms through which social anxiety increases smartphone addiction vulnerability, specifically via the mediating role of loneliness.

### Hypothesis testing and theoretical implications

The results supported Hypothesis 1, indicating that social anxiety exerted a positive predictive effect on smartphone addiction, which aligns with prior research (32). This pattern aligns with the I-PACE model’s proposition that affective dysregulation drives technological dependency (16). Social anxiety sufferers often exhibit excessive focus on self-expression, preset negative outcomes, and selectively focus on negative signals during face-to-face interactions. At the same time, They tend to anticipate awkward social situations and overestimate the likelihood of negative evaluation, making them more inclined to maintain a “safe distance” through excessive smartphone use (80). Heightened social anxiety leads to avoidance of real-world social engagement, prompting compensatory smartphone use to meet social needs (81). However, this coping strategy can paradoxically increase the risk of smartphone addiction by reinforcing a cycle in which temporary relief from anxiety encourages more screen time (82).

Our findings revealed that social anxiety positively predicted loneliness, which in turn positively predicted smartphone addiction. This suggests that socially anxious individuals exhibit significantly elevated loneliness susceptibility, which subsequently increases their risk of smartphone addiction. These results supported Hypothesis 2. Consistent with previous research (83), college students with social anxiety symptoms report significantly higher levels of loneliness. Specifically, socially anxious individuals often experience social fear, heightened discomfort, and somatic symptoms (e.g., tremors, blushing, and palpitations) from the anticipation of social interactions until their conclusion. These symptoms frequently lead to anticipatory anxiety and post-interaction rumination. Such affective and physiological responses drive avoidance behaviors, which substantially restrict opportunities for meaningful face-to-face social engagement (84). Consequently, this behavioral pattern reduces relationship formation and maintenance, further increasing vulnerability to loneliness.

Prior research has shown that individuals with social phobia typically have fewer social relationships than the general population (84). Individuals with social anxiety disorder often face challenges in various aspects of social functioning. Research has shown they tend to report fewer close friendships, less frequent peer interaction, and greater difficulties in forming romantic or emotionally intimate relationships compared to peers without social anxiety (85–89). According to Social Disconnection Theory, diminished social connections and impaired interpersonal communication could exacerbate feelings of loneliness (90). The I-PACE model posits that individuals experiencing negative affect may engage in maladaptive smartphone use as a compensatory strategy to alleviate emotional distress (16). In this context, the ubiquitous availability and multifunctional nature of smartphones make them particularly appealing to young adults, who often turn to entertainment-based smartphone use as a means of mitigating loneliness (91, 92). However, when such usage escalates beyond self-regulation, it may transition into problematic behavior characterized by loss of control and increased dependency (17).

In addition, this study observed that both the correlation between gender and social anxiety (*r* = 0.058, *p* < 0.01) and that between age and loneliness (*r* = 0.046, *p* < 0.05) were below 0.1. Despite statistical significance, these associations exhibited negligible effect sizes (Cohen’s criteria for small effects: *r* < 0.1; 1988), suggesting that demographic variables exerted minimal predictive utility for the outcome variables. This paradoxical pattern of “significant yet weak” correlations might be an artifact of the large sample size (N = 2113), which amplifies statistical sensitivity to even trivial effects. Given that demographic variables were not central to the study’s hypotheses, these findings serve solely as descriptive annotations of sample characteristics and do not compromise the validity of the primary conclusions.

### Implications for practice

This study highlights that social anxiety and related states (such as loneliness) are key responses in understanding smartphone addiction. Social anxiety may intensify psychological distress and negative emotions, thereby precipitating a functional transition of smartphones from utilitarian tools to maladaptive vehicles for emotional soothing (93). While smartphone use can serve as self-therapy for negative emotions (94), overreliance on this coping strategy may result in functional impairments (16).

Given the established psychosocial linkages among social anxiety, loneliness, and smartphone addiction, this study advocates for multilevel prevention frameworks wherein university administrators integrate mental health literacy into campus wellness programs, while parents should encourage college students to engage in face-to-face social interactions to disrupt the maladaptive cycle of digital dependency. So, the university superintendents and teachers should take some practical and effective measures to alleviate or eliminate social anxiety among college students, including assessing social anxiety (95), the development of communication capacity education (96), cognitive behavioral therapy (97). In recent years, mindfulness has demonstrated notable promise in managing addiction and psychological challenges, with its application in addressing loneliness and smartphone addiction particularly drawing substantial scholarly attention (98–102). Thus, college teachers are recommended to incorporate mindfulness training into college student learning to suppress the negative impact of social anxiety on loneliness and smartphone addiction (103).

### Limitations and future directions

Several limitations of this study warrant acknowledgment. First, the cross-sectional design precludes causal inference, necessitating longitudinal designs in future research to establish temporal precedence among variables. Second, reliance on self-report measures introduces potential social desirability bias and other response artifacts, which could attenuate validity. Future investigations might triangulate data using multiple informants (e.g., peers, teachers) and objective measures (e.g., smartphone usage logs) to enhance reliability. Third, the single cultural context (China) and homogeneous sample (college students) restrict generalizability. Replication in diverse populations (e.g., cross-national samples, non-student groups) would strengthen external validity. Furthermore, this study did not incorporate variables related to smartphone usage patterns (such as social interaction, instrumental, and recreational usage), university environmental factors (academic stress, campus social support), and family background (parenting styles, parent-child attachment). The omission of these variables may hinder a comprehensive understanding of the mechanisms underlying the influence of social anxiety on smartphone addiction among college students. Specifically, it limits the ability to elucidate the mediating or moderating roles of environmental factors, thereby constraining the real-world explanatory power of the study’s conclusions. Therefore, future studies should address these limitations by adopting more comprehensive research designs and incorporating a wider range of variables to gain a deeper understanding of the complex relationships involved. By doing so, we can further refine and strengthen the I-PACE model, ultimately contributing to more effective interventions for smartphone addiction among college students with social anxiety. Notwithstanding its limitations, the research advances understanding of the psychological mechanism linking social anxiety to smartphone addiction and provides preliminary evidence for loneliness as a partial mediator. Findings have practical implications for developing targeted interventions to address problematic smartphone use in socially anxious populations.

## Conclusion

This study investigated college students through structural equation modeling to delineate the effects of social anxiety and loneliness on smartphone addiction. Social anxiety is significantly correlated with smartphone addiction, and loneliness partially mediating their relationship. Reducing loneliness can prevent smartphone addiction among college students with social anxiety. These findings provide additional empirical support for the I-PACE model.

## Data Availability

The raw data supporting the conclusions of this article will be made available by the authors, without undue reservation.
